# Stereotypes of Older Adults, Older Men, and Male Leaders Predict Expectations, Stance, and Voting Intentions

**DOI:** 10.1093/geroni/igab046.3640

**Published:** 2021-12-17

**Authors:** Caitlin Monahan, Ashley Lytle, Elizabeth Inman, Marybeth Apriceno, Jamie Macdonald, Sheri Levy

**Affiliations:** 1 Stony Brook University, Brooklyn, New York, United States; 2 Stevens Institute of Technology, Hoboken, New Jersey, United States; 3 Stony Brook University, Stony Brook University, New York, United States; 4 Stony Brook University, Stony Brook, New York, United States; 5 St. Francis College, Brooklyn, New York, United States

## Abstract

The 2020 U.S. Presidential Election offered a unique opportunity to examine how stereotypes of older adults, older men, and male leaders impact expectations of candidate job performance and intentions to vote for Biden or Trump. This online study involved 500 college students from two universities from September 30th until November 3 (Election Day). A Biden and Trump model were tested for the relationships among (a) stereotypes from public discourse with (b) expectations of candidates/ presidential performance with (c) voting stance (pro- and anti-Biden vs pro- and anti-Trump) and (d) intentions to vote for Biden/Trump. As expected, for the Biden model, endorsement of older adult (lesser endorsement of senile, unhealthy), male leadership (greater endorsement of assertive and collaborative, lesser endorsement of uncaring), and older male stereotypes (greater endorsement of elder statesman and family-focused) predicted greater expectations of Biden’s performance, which predicted pro-Biden and anti-Trump stances and ultimately voting intentions for Biden. As expected, for the Trump model, endorsement of older adult (lesser endorsement of senile), male leadership (greater endorsement of assertive, collaborative, lesser endorsement of immoral and uncaring), and older male stereotypes (greater endorsement of elder statesman) predicted greater expectations of Trump’s performance, which predicted pro-Trump and anti-Biden stances and ultimately voting intentions for Trump. Taken together, these results suggest examining relevant categories of stereotypes associated with candidates and voting stances provides a fuller picture of voting behavior toward multiple candidates vying for office in addition to political ideology and voting intentions.

